# Generative AI (gAI) in medical education: Chat-GPT and co

**DOI:** 10.3205/zma001636

**Published:** 2023-06-15

**Authors:** Sören Moritz, Bernd Romeike, Christoph Stosch, Daniel Tolks

**Affiliations:** 1 University of Cologne, Medical Faculty, Cologne, Germany; 2 University Medical Center Rostock, Academic Dean's Office, Division of Medical Education, Rostock, Germany; 3 Leuphana University Lüneburg, Centre for Applied Health Promotion, Lüneburg, Germany

## Editorial

“The use of chatbots in medical education is an emerging trend that is welcomed by many educators and medical professionals. In particular, the use of ChatGPT, a large language model of OpenAI, offers a variety of benefits for students and educators alike […]” [1]. So far so amazing, the passage already points to the whole dilemma: will teaching at universities ever be the same after ChatGPT as it never was anyways? 

We had a Cologne term paper in the “field of competence carcinogenesis” (interdisciplinary teaching in the first preclinical study semester) generated in triplicate by ChatGPT, each with identical queries, and received three different two-page texts including literature citations according to APA style. These have been examined by two detector programs (Groover, Writer) to determine whether they were written by a human or a bot. Both programs could not detect them as machine-written (cave: short texts are practically undetectable). The search for plagiarism with the software PlagAware did not reveal any conspicuous passages worthy of consideration (approx. 3-5% agreement with already published texts). The papers were forwarded unchanged to the assessing tutors with the result that two papers were assessed as “passed” and one as “failed”. The poor performance was due to certain terms used in the field of competence that was not named, as well as a non-matching literature citation. What next? 

Let’s ask ChatGPT: “...If students were able to access ChatGPT and ask questions during the exam, they could theoretically receive answers from ChatGPT that could help them answer exam questions...” [2].

### What is the consequence?

1. *Assessments in presence:* With adequate assessment supervision, ChatGPT does not pose a danger in terms of an increase in cheating in written, oral and practical assessment in presence.
2. *Homework and presentations:* Since most of the assessments, especially in medicine, are conducted in the form of MCQs in presence, the assessment formats “homework” or “presentation” are negligible. Nevertheless, existing, purely written, non-supervised assessment formats will have to be questioned. The assessment format “presentation” could be still used if the free presentation of scientific contexts is the focus. Here, the teacher has the opportunity to also assess the degree of mastery of the subject matter as the core aspect of the oral exam.
3. *Final theses (Bachelor, Master), scientific projects (as required by the new ÄApprO), dissertation papers:* Here a still unsolved problem of possible deception arises, since written texts and thoughts don’t represent own achievements, but could be generated by AI. The oral assessment (i.e., the doctoral colloquium, etc.) has an increasingly important function.

Are the implications for the assessment culture at the university the only problem for universities, or what other implications arise from using generative AIs? To do this, one needs to understand, at least in principle, how such a generative AI works?

To this end, ChatGPT say: “ChatGPT is a so-called “language-model-AI” or “Natural Language Processing” (NLP) AI based on deep learning. It has been trained with large amounts of human text, allowing it to generate and understand human-like text. To generate texts, ChatGPT uses a neural network that consists of many layers and has millions of parameters. It works like a very complex pattern recognition system that is able to recognize contextual relationships between words and sentences. For example, when you ask ChatGPT a question or type a sentence, the model tries to generate an appropriate answer or continuation based on what it has learned from its training data. [...] It is important to emphasize that AIs like ChatGPT are just tools and that their use is the responsibility of the people who use them. It is up to us as a society to ensure that AIs are used in ways that are both effective and ethical.“ [3].

In summary, ChatGPT (GPT 3.5) models human speech better than any other computer program before it. It can simulate a real conversation partner and help answer complex facts and questions surprisingly effectively. 

The latest version of OpenAI’s language models is called GPT-4. It was released on March 14, 2023 and is capable of generating more accurate and reliable statements than its predecessors GPT-3 and GPT-3.5. Another advantage is that GPT-4 is multimodal and can accept images as input. It can generate image captions, classifications, and analyses. The capabilities of GPT-4 are impressive, especially in exams: In a simulated BAR test, GPT-3.5 scored in the bottom 10% percentile, whereas GPT-4 performs in the top 10% percentile [4].

By using ChatGPT and similar AI tools, open-ended questions arise at multiple levels:

- Even if questions can be answered by generative AIs (gAI) in a predominantly factually accurate manner, who is responsible for the application and use of this knowledge? How does the accountability of decision makers change, for example, at the bedside or in teaching?
- What are the legal implications of the use of gAI on areas of law such as copyright (plagiarism), liability (malpractice), or corporate law (business models)?
- What impact do gAIs have on diversity or equity?
- In the long run, will gAI replace certain professions (dermatologists, pathologists, radiologists) or the journals (e.g., the JME), or will gAI give “superpowers” to some professions?
- Is there a risk of reinforcing educational inequity by students and institutions who have access to the tools and the skills to use them and those who don’t [5]?
- How will the authenticity of digital information be ensured in the future and how will this impact our media literacy?
- How do we design digital, university learning and assessment processes so that students become academic experts and use various gAIs in a productive way?
- How does teaching and learning change when lecturers have instructional materials created by gAI?
- What are the consequences of using gAI on communication with colleagues, patients, or on reflection?
- There is also the question, at which point the illegal use starts at all. The spell checker of Word and Grammarly® is used ubiquitously and is mostly recognized as an appropriate tool. But at what point is a paraphrase really plagiarism? The “prompt” comes from the author and mostly the generated text is further adapted if necessary.

Let’s turn the tables for once, because the AI will burn itself into the monitor, and yet the universities and consequently the teaching will continue to exist: Has the vinyl record disappeared, even though there were first CD’s and now streaming? At least there has been an amazing renaissance for vinyl records since 2010 [6]. However, in the face of climate change and resource scarcity, we will very soon be limited to purely digital formats, precisely because everyone will generate professional music themselves through “whatever-to-music” AI converters and share it via social media. Putting it positively, could gAIs be helpful in teaching? 

Active use of gAIs in teaching: statements about specific, medical knowledge contexts generated interactively by students using gAIs can be analyzed by them and help to train higher cognitive functions such as “evaluation and assessment” (according to Bloom [7]) and thereby get a good overview of the topics. Students are forced to change roles in this process. They are the authors of the “prompts”, receive writing support from gAI and then, however, have to prove their expertise as editors and evaluate the generated texts or correct them as well as possible. But: How do students “climb” to the higher levels according to Bloom, if knowledge acquisition with a bot works fundamentally different? 

Via the use of gAIs, students can practice asking the right questions, a core competency of Evidence Based Medicine. During the development of research questions the chatbot can help sharpen and delineate them. It can also suggest different methods and study designs. The gAI is helpful and efficient in paraphrasing texts. 

In medicine, training in problem-solving skills (clinical decision making) is an important competence. Here, the so-called background knowledge probably plays a central role. This background knowledge can be specifically improved by the application of gAI, in which gAI expands the views on the problem (“chatbot PbL”?). In the end, we get some differential diagnoses added that we just didn't think of. 

gAI will establish itself as an interactive reference work. It is to be expected that the use of AI on the wards will be standardized with an evidence-based approach (GPT-4). The use of these tools will then be expected as part of physician competence and, to that extent, will also need to be trained (replaces gAI the German-language test preparation software AMBOSS?).

gAI can be used to create virtual patient cases. On the one hand, the linguistic quality can be improved and on the other hand, more exciting cases can be generated, for example with the help of storytelling elements or paraphrasing.

gAI can be used to create storyboards or stage directions for educational films.

gAI can support the enforcement of competence-based forms of assessment: Alone the use of the available information for the benefit of the patient*s (i.e. the competence in dealing with the counterpart) should increasingly be the subject of new assessment scenarios (workplace-based assessment, simulations, physical examinations, oral assessments, ...).

There are first experiences that on the lecturer side gAI can be used well for the creation of MC questions: Here, it is especially about the targeted search for distractors.

### Conclusion

The emergence of generative AI tools is a game changer that some experts compare to the introduction of the smartphone. Given the remarkable advances seen in recent months, as well as those expected this year, it’s fair to say that the impact on humanity will be as significant as the displacement of horses from cities with the introduction of the automobile. Unlike cars, which took a century to evolve into the sophisticated machines we have today, generative AI tools need only the year 2023 for a significant transformation, resulting in a highly notable impact. Those who embrace it will have a clear advantage over those who don’t. 

There needs to be a clear strategy within our education system to keep up with the rapid evolution of gAI tools and to continuously integrate them into our curricula and syllabi. The opportunities and risks of gAI on our teaching and learning need to be continuously analyzed so that didactic strategies can be adapted in a timely manner. We are likely to run somewhat behind developments in the coming months and years. Therefore, it seems all the more important to consistently conduct evidence-based teaching research in all integrations of gAIs into our teaching:

In this sense, the authors of the editorial would like to invite you to use the summer semester to engage in experiences with generative AI in teaching and learning at universities, and to bring these thoughts, preliminary studies, and experiments to a bar-camp at the upcoming annual meeting of the Society for Medical Education in Osnabrück [https://gma2023.de/] as a contribution. Welcome Industrialization 5.0. 

A collection of links on the topic of generative AI is available at: [https://padlet.com/danieltolks1/linksammlung-didaktik-und-chatgpt-ausschusss-digitalisierung-ihupnj9wb3y0foz3]

### More articles in this issue

In their study, Gisi et al. examined the objective and subjective effects of pandemic-related changes in the course of studies on the perception of the practical year [8]. In their study, Brütting and colleagues took a closer look at the factors conducive to working as a doctor in rural regions and the awareness of districts in the catchment area of universities [9]. In an intervention study, Hopp et al. examined the influence of teaching medical students close to patients on the stigmatization of people with mental illness [10]. In their study, Kiesewetter and colleagues were able to demonstrate a positive influence in the use of dogs in therapy with children with rheumatic diseases [11]. Kruse et al. present the results of a survey on learning strategies of dental students [12]. Mand et al. present the implementation of a multi-stage observer training for medical students to assess simulated pediatric emergency situations [13]. Jannik Osten and colleagues deal with the question of whether face-to-face lectures are still up-to-date and whether synchronous online lectures are an alternative [14]. In their questionnaire survey, Daunert and colleagues investigated the question of what motivates GPs to train medical students in their practice [15]. Dasci et al. evaluated different forms of training in knotting and suturing techniques in a controlled randomized study in dentistry [16]. The working group Quality Management in Education, Training and Continuing Education of the Society for Quality Management in Health Care (Gesellschaft für Qualitätsmanagement in der Gesundheitsversorgung e.V., GQMG) has published a position paper on the topic of quality management in medical studies in the competence-based learning objectives catalogue [17]. Koch et al. describe the development of an interactive elective “modified anatomy” for students within the framework of the Z-curriculum according to NKLM 2.0 [18]. Laura Wortmann et al. present the results of a survey on the topic of gender medicine in teaching [19].

## Competing interests

The authors declare that they have no competing interests. 
